# An evaluation of additives for mitigating the risk of virus‐contaminated feed using an ice‐block challenge model

**DOI:** 10.1111/tbed.13749

**Published:** 2020-08-06

**Authors:** Scott A. Dee, Megan C. Niederwerder, Roy Edler, Dan Hanson, Aaron Singrey, Roger Cochrane, Gordon Spronk, Eric Nelson

**Affiliations:** ^1^ Pipestone Applied Research Pipestone Veterinary Services Pipestone Minnesota USA; ^2^ Department of Diagnostic Medicine/Pathobiology College of Veterinary Medicine Kansas State University Manhattan Kansas USA; ^3^ Department of Veterinary and Biomedical Sciences South Dakota State University Brookings South Dakota USA

**Keywords:** animal feed, feed mitigation, ice‐block challenge, swine viral diseases

## Abstract

The role of animal feed as a vehicle for the transport and transmission of viral diseases was first identified during the porcine epidemic diarrhoea virus (PEDV) epidemic in North America. Since that time, various feed additives have been evaluated at the laboratory level to measure their effect on viral viability and infectivity in contaminated feed using bioassay piglet models. While a valid first step, the conditions of these studies were not representative of commercial swine production. Therefore, the purpose of this study was to evaluate the ability of feed additives to mitigate the risk of virus‐contaminated feed using a model based on real‐world conditions. This new model used an ‘ice‐block’ challenge, containing equal concentrations of porcine reproductive and respiratory syndrome virus (PRRSV), Senecavirus A (SVA) and PEDV, larger populations of pigs, representative commercial facilities and environments, along with realistic volumes of complete feed supplemented with selected additives. Following supplementation, the ice block was manually dropped into designated feed bins and pigs consumed feed by natural feeding behaviour. After challenge, samples were collected at the pen level (feed troughs, oral fluids) and at the animal level (clinical signs, viral infection, growth rate, and mortality) across five independent experiments involving 15 additives. In 14 of the additives tested, pigs on supplemented diets had significantly greater average daily gain (ADG), significantly lower clinical signs and infection levels, and numerically lower mortality rates compared to non‐supplemented controls. In conclusion, the majority of the additives evaluated mitigated the effects of PRRSV 174, PEDV and SVA in contaminated feed, resulting in improved health and performance.

## 1 INTRODUCTION

Effective biosecurity protocols are essential towards protecting the health status of swine farms. In the United States, significant resources have been invested to reduce the risk of viral pathogens, such as the entry of porcine reproductive and respiratory syndrome virus (PRRSV) into susceptible populations. Protocols including shower in–shower out of personnel, transport sanitation, quarantine and testing of incoming genetics, and the filtration of incoming air are commonplace, particularly at the level of the sow farm (Silva, Corbellini, Linhares, Baker, & Holtkamp, 2018). In contrast, prior to introduction of porcine epidemic diarrhoea virus (PEDV) into the US national swine herd (Chen et al., 2014), the role of feed as a vehicle for pathogen transport and transmission had not been considered, despite the fact that feed is delivered to swine farms on a daily basis in the absence of a biosecurity plan. Following proof of concept of the transmission of PEDV to pigs following natural consumption of contaminated feed (Dee, Clement, et al., 2014), information on the oral infective dose of PEDV in feed (Schumacher et al., 2016) and demonstration of the widespread PEDV contamination of surfaces in an animal feed manufacturing facility (Schumacher et al., 2017) was published. Shortly thereafter, data on the survival of multiple viruses, including PEDV, PRRSV, Senecavirus A (SVA) and African swine fever virus (ASFV) in feed ingredients during simulated trans‐Pacific and trans‐Atlantic shipments, became available (Dee et al., 2015, 2016, 2018). Finally, recent studies demonstrating the transmission of ASFV via contaminated feed, along with the calculation of the half‐life (T ½) of ASFV in feed ingredients, have raised the awareness of feed as a potential vehicle for viral transport and transmission (Niederwerder et al., 2019; Stoian et al., 2019, 2020).

In an effort to manage this risk, follow‐up studies evaluated the effect of various additives on viral‐contaminated feed (Cochrane et al., 2019; Dee, Neill, Clement, Christopher‐Hennings, & Nelson, 2014; Huss et al., 2017; Trudeau et al., 2016). The initial work focused on PEDV and SalCURB^®^ (Kemin Industries), an FDA‐approved formaldehyde‐based liquid antimicrobial to control *Salmonella* contamination in poultry and swine diets. In groups of pigs fed PEDV‐positive feed treated with SalCURB^®^, clinical signs of porcine epidemic diarrhoea were not observed (Dee, Neill, et al., 2014). In another study, feed samples spiked with PEDV and mixed with either organic acids, sugar or salt resulted in a reduction in PEDV survival (Trudeau et al., 2016). Finally, the addition of medium‐chain fatty acid blends to PEDV‐contaminated feed significantly reduced viral RNA and infectivity (Cochrane et al., 2019).

While these data were promising, a significant limitation of all studies was that they were conducted under artificial conditions, involving small volumes of feed, and laboratory‐derived 1–2 ml inoculums that were manually delivered per os to individual baby piglets. To increase industry confidence in the use of additives to mitigate the risk of viral‐contaminated feed, experiments needed to be scaled‐up in size and scope, incorporating conditions representative of commercial swine operations, that is larger populations of animals, manufacturing, storage and volume of feed, natural feeding behaviour, a more robust viral challenge, and a wide range of products with diverse chemistries. Therefore, the purpose of this study was to design a challenge model that involved commercial conditions to test the ability of multiple additives to prevent disease transmission through feed. The study was based on the hypothesis that in the presence of virus‐contaminated feed, the use of additives would significantly improve pig performance, as compared to pigs on non‐mitigated diets, in the face of viral challenge.

## 2 MATERIALS AND METHODS

### 2.1 Animal care and use

The overall study period was scheduled for November 2018 through February 2020. It was designed to include five independent experiments involving 2,880 weaned pigs, ranging from 5 to 8 weeks of age on arrival, based on the availability and cost. Throughout the entire study period, animals were managed in accordance with the institutional animal care and use guidelines observed by the investigators' ethical review board (Pipestone Applied Research IACUC trial numbers 2018‐5, 2019‐10 and 2020‐02).

### 2.2 Experimental design

Across all five experiments, room was the experimental unit with pens within the room serving as replicates. For the five experiments, 576 pigs from a swine herd negative to PRRSV, PEDV and SVA were allocated equally across the six rooms of the Pipestone Applied Research biosafety level 2 facility. Each room contained six pens with 16 pigs per pen, and each room was assigned a designated feed bin. A ‘treatment group’ was a room of animals fed complete feed supplemented with a specific product, while a ‘positive control group’ consisted of a room of animals fed complete feed in the absence of supplementation. Each experiment was designed to be 25 days in length, involving a 10‐day pre‐challenge period which allowed animals to acclimate to their surroundings and respective diets, followed by a 15‐day post‐challenge period to measure response. During the 15‐day post‐challenge period, it was planned to purposefully contaminate the feed in each bin on day 0 and day 6, and to collect samples on days 0, 6 and 15. On day 15 post‐challenge, the experiment was terminated, final samples collected, and all animals humanely euthanized via captive bolt.

### 2.3 Description of facility and personnel biosecurity

The facility used in the study was a certified biosafety level 2 building. To enter the facility, personnel showered in using the entry shower, donned barn‐specific clothing and footwear, and moved to the individual animal rooms via a designated entry (clean) hallway. Each room had a separate ventilation system that filtered air in and out of each room (MERV 4 pre‐filters plus MERV 16 filters, 3M, St Paul, MN, US), a separate slurry pit of 1.2 m in depth, and a Danish entry system in the anteroom, where personnel donned room‐specific coveralls, boots and gloves. Upon exiting each room, personnel removed room‐specific coveralls, boots and gloves, and left the animal airspace via an exit chamber and waited 2 min to purge air from the room prior to opening the exit door. Personnel walked to an exit shower via a designated exit (dirty) hallway and showered again. This procedure was repeated between every room, and rooms were visited in numerical order every day (rooms 1–6) before one final shower was taken prior to exiting the facility.

### 2.4 Selection of additives

Since an objective of the overall project was to test a wide range of additives with diverse chemistries, it was important to recruit multiple products. Identification of potential candidates took place through conversations with the American Feed Industry Association, and stakeholders from commercial companies. All participants provided funds to cover the costs for their portion of the trial, along with the necessary product.

### 2.5 Preparation of animal feed and quality control of application

For each of the five experiments, it was determined that a 1.3 metric ton batch of feed would be needed for each room, based on 96 animals per room, an estimated daily feed intake of 0.57 kg, and a feeding period of 25 days. Across all five experiments, rooms 1–5 of the facility were designated as treatment rooms and room 6 as the positive control room. Product application (non‐formaldehyde‐based) occurred at commercial milling operations (Chandler Feed), in Chandler, MN and Leota, MN), and while the application of formaldehyde‐based products took place at Lester Feed and Grain, Lester, IA. Prior to delivery to the research facility, feed (with the exception of the positive control) was supplemented with products at a pre‐determined inclusion rate(s) at the mills. Oversight of the mixing process was conducted by Pipestone research personnel and a company representative. Feed was then delivered to the research facility into a specific bin, according to room designation.

### 2.6 Preparation of ice‐block challenge material

Feed contamination (challenge) was accomplished using a 500 ml block of ice containing equal concentration of PRRSV‐174, PEDV and SVA in minimal essential medium (MEM) (Sigma Aldrich). To prepare an ice block, each virus stock was diluted in 100 ml of MEM to a concentration of 1 × 10^5^ 50% tissue culture infectious dose/mL (TCID_50_/ml), resulting in 100 ml of PEDV, 100 ml of SVA and 100 ml of PRRV‐174. Following mixture of the viruses, 200 ml of MEM was added to each ice block to bring the total volume to 500 ml/block to deliver a concentration of 1 × 10^7^ 50% tissue culture infectious doses of each virus into 1,300 kg of complete feed, resulting in a concentration of 7.69 v 10° 50% tissue culture infectious doses per gram of feed. Blocks were frozen in plastic containers of 177 mm × 124 mm × 56 mm in size (Matchups, Décor Corp, Melbourne, Australia) and stored at −80°C until the designated days of feed contamination. Feed contamination (challenge) occurred on days 0 and 6 of each experiment, when an ice block was manually dropped into the bin through its opening at the top. Feed was then delivered as needed into each animal room via the auger system and consumed by natural feeding behaviour.

### 2.7 Sample collection and diagnostic testing

The sampling programme was designed to answer three questions:

1. *Did the viruses enter the room via feed?* To answer this question, feed samples were collected from the feed trough.
2. *Were pigs exposed to the viruses?* To answer this question, oral fluid samples were collected.
3. *Did the pigs get infected?* To answer this question, a subset of pigs were necropsied and evaluated for the presence of virus in select samples.

Throughout the 15‐day period post‐challenge, samples were collected on day 6 and day 15. On these days, samples from the two feed troughs in each pen and oral fluid samples from each pen were collected. For collection of feed trough samples, personnel wore disposable gloves, applied a single dry pad (Swiffer, Procter and Gamble) to both feed troughs in each pen and inserted it into a plastic bag (Ziploc, Dow Chemical Company). The cloth was immersed in 10 ml of sterile saline (American Pharma Wholesale), manually squeezed into the bag, and 3 ml was decanted into a plastic snap cap tube (Falcon, Becton Dickinson). For collection of oral fluid samples, a single cotton rope was hung in each pen and processed as published (Prickett & Zimmerman, 2010). All samples were sent to the South Dakota State University Animal Disease Research and Diagnostic Laboratory (SDSU ADRDL) for PCR testing.

On day 15 post‐challenge, 30 of the 96 animals from each room were selected for necropsy. Selection was purposefully biased to choose animals demonstrating clinical signs suggestive of PED (diarrhoea) or SVA (lameness) or PRRS (dyspnoea). If clinical signs were not observable, substandard animals (runts, lightweight culls, etc.) were selected. If the room contained only clinically normal, high quality pigs random sampling was conducted until 30 animals were identified. For determination of PRRSV infection, serum samples were collected from the 30 animals and pooled 5:1. For determination of PEDV infection, rectal swabs were collected from each pig. For determination of SVA infection, tonsil samples were collected from each pig and tested individually. All samples were tested by PCR using standard procedures employed at the SDSU ADRDL (Dee, Clement, et al., 2014).

### 2.8 Clinical signs and animal performance

On days 6 and 15, the six pens in each room were evaluated for clinical evidence of diarrhoea (PEDV), lameness (SVA) or dyspnoea (PRRSV). If one or more pigs in a pen demonstrated any of the aforementioned signs, it was recorded as a single event. For example, if one or more pigs in a pen were demonstrating clinical signs of diarrhoea on day 6 and again on day 12, the pen was only counted once. At the end of each experiment, the number of pens with diarrhoea, lameness or dyspnoea in each room was summed according to clinical sign and divided by six (number of pens per room) to calculate the percentage of affected pens exhibiting each clinical sign. On days 0 and 15, pen weights from each room were collected, along with the number of mortalities during the post‐challenge period.

### 2.9 Facility sanitation and monitoring

Between each experiment, all rooms were sanitized and surfaces were monitored for the presence of viral RNA. The sanitation protocol involved disposal of garbage and remaining supplies. Clothing and boots were removed and sanitized. Rooms were first washed using 82.2°C water to remove all visible organic material. A cleaning agent containing a 7.5% alkyl dimethyl benzyl ammonium chloride (Ag Forte Pro, Atmosphere Global) was applied to surfaces using a hydrofoamer (Hydro Systems) to remove biofilm. Rooms were disinfected once using a product composed of 26% alkyl dimethyl benzyl ammonium chloride and 7% glutaraldehyde (Synergize, Neogen), and twice again with a 6% sodium hypochlorite product (Clorox, The Clorox Company) via a hydrofoamer. Specific surfaces, such as filter banks, water cups, feed troughs, feed motors, electrical conduit and the surfaces within the entry and exit chambers were hand wiped (Clorox wipes, The Clorox Company) and floors were scrubbed. Disinfectant water accumulated in the slurry pits and after cleaning was completed, were drained to an external concrete‐covered lagoon. Rooms were heated to 21°C and sat empty for seven days. In addition, feed bins, feed boots and feed lines were emptied and residual dust removed using compressed air. An 8.16 kg quantity of a dry 5.25% calcium hypochlorite foot pan powder (Traffic C.O.P, Paragon Specialty Products) was added to each empty bin while feed lines were in operation. This was followed by the addition of 68.2 kg of ground corn to each bin, with lines allowed to run until all corn had been transferred from bins to the feeders. Feeders were then emptied and sanitized as described. Finally, pre‐filters were replaced on both the incoming and outgoing filter banks. After rooms had sat empty for seven days, surfaces in each room and the feed system were sampled using Swiffer cloths (as described). Designated sampling areas collected included corn samples from feeders following flushing, feed troughs, water cups, pen gating, feed line tubing, auger motors, and electrical wiring conduit, underneath the slotted flooring and between metal slats, room heaters and fans, flooring of entry and exit anterooms, the anteroom sink area, soles of all boots, garbage cans, and assorted equipment contacting pigs such as cable snare, pig panels and paddles. The samples were processed (as described) and tested for the presence of PEDV RNA, PRRSV RNA and SVA RNA by PCR at the SDSU ADRDL. If any samples were positive, problem areas were re‐sanitized and re‐tested. A room was not considered ‘clean’ until all samples were PCR‐negative, and no experiment commenced without the entire facility (all six rooms) and the feed system testing clean.

### 2.10 Data analysis

Average daily gain was analysed using one‐way analysis of variance (ANOVA) where room was considered the experimental unit with pens within room as replicates. When the f‐statistic was less than 0.05, Tukey–Kramer HSD was used to discern differences amongst the treatment groups with alpha ≤ 0.05. Clinical scores and post‐mortem samples organized as rates were analysed using Pearson's chi‐square test. When the *p*‐value was ≤.05, further pairwise comparisons were made using Fisher's exact test to discern differences amongst the treatments. In addition, a meta‐analysis of ADG was computed across all five experiments using the raw mean difference approach (fixed effects model), as all experiments were conducted and reported using the same general design and outcome scale. The reported average daily gains from each of the mitigated treatment groups were averaged and subtracted from the average daily gain for the positive control groups from each of five respective mitigation studies to arrive at a raw mean difference. A forest plot (Comprehensive Meta‐Analysis, Englewood, NJ) was constructed outlining the difference in the means, standard errors, 95% confidence intervals and *p*‐values from the five experiments. The summary effect reports the weighted average difference, standard error, and 95% confidence interval, and *p*‐value across all five experiments.

## 3 RESULTS

### 3.1 Products tested

Across the entire study period, 12 companies participated in the trial, resulting in the testing of 15 products with four tested at different inclusion rates (Table 1).

**Table 1:** Summary of feed additives tested during the study, including product name, company, description of ingredients and inclusion rate

Product	Company	Description	Inclusion rate
DaaFit^®^S	ADM	A source of fatty acids, including lauric and myristic acids and glycerol monolaurate	0.5% or 0.3%
DaaFit^®^PLUS	ADM	An acidifier blend composed of short‐chain fatty acids, formic, propionic acid, acetic acid, sorbic acids and a blend of medium‐chain fatty acids including lauric acid, caprylic acid, and glycerol monolaurate	0.5%
Guardian	Alltech	A blend of organic acids and essential oils	0.44%
pHorce	Anpario	A blend of liquid formic and propionic acids on a mineral carrier	0.3%
VVC	DSM	Pure benzoic acids with nature‐identical flavourings	0.5% or 0.3%
FINIO^®^	Anitox	A blend of propionic acid, trans‐2‐hexenal (leaf aldehyde) and nonanoic acid (pelargonic acid)	0.2%
SalCURB^®^	Kemin	A blend of aqueous formaldehyde and organic acids	0.275%
CaptiSURE™	Kemin	Medium‐chain fatty acid blend	0.5% or 1.0%
SalCURB^®^K2	Kemin	An organic acid blend, including formic acid, ammonium formate, propionic acid and lactic acid	0.275%
FURST PROTECT	McNess	A blend of emulsifying monoglycerides of medium‐chain fatty acids and essential oils plus botanical extracts	0.4%
Activate DA	Novus	A blend of organic acids and methionine hydroxy analogue (HMTBa)	0.5% or 0.15%
Dominnate	Purina Animal Nutrition	A blend of 3 medium‐chain fatty acids	0.5%
Dual Defender™	Ralco	A blend of essential oils and prebiotic fibre	0.1%
R2™	Feed Energy	A natural lipid‐based line of products made by a combination of short‐, medium‐ and long‐chain fatty acids	3.0%
Vigilex	Provimi	A blend of oils, bacterial fermentation products, whey products, plant protein and natural flavourings	0.4%

### 3.2 Experiment 1 summary

Experiment 1 was conducted in November 2018. This experiment involved a single product, Activate DA, a blend of organic acids and methionine hydroxy analogue (HMTBa) (Novus International) at two different inclusion rates per ton of feed: 0.5% (per label) and a reduced dose (0.15%), along with a positive control group. Based on data summarized in Table 2a, PRRSV, PEDV and SVA infection occurred post‐challenge in the positive control group, along with clinical signs suggestive of all three diseases, poor performance (0.65 kg ADG) and elevated mortality (4%). Across the metrics evaluated, the health and performance of animals consuming the 0.5% level of Activate DA was significantly different as compared to the positive control group, with an average daily gain improvement of 115% (0.75 kg versus 0.65 kg, *p* < .0001) along with significantly lower clinical scores. In regard to the two treatment groups, ADG was significantly greater in the 0.5% group versus the 0.15% group (0.75 kg versus 0.64 kg, *p* < .0001). Clinical signs were not observed in either treatment group, and no evidence of SVA or PRRSV RNA was detected in tonsil or serum samples. While PEDV RNA was detected in rectal swabs from both treatment groups, a significantly lower number of PEDV‐positive rectal swabs (7/30 versus 28/30, *p* < .0001) were observed in the 0.5% group. Finally, PRRSV, PEDV and SVA RNA were detected in multiple feed trough and oral fluid samples from the positive control group; however, only PEDV and SVA RNA were detected in feed trough samples and oral fluid samples from the treatment groups (Table 2b).

**Table 2a:** Summary of clinical scores, post‐mortem diagnostics and pig performance from Experiment 1

Treatment	Clinical scores			Post‐mortem diagnostics			Performance	
	Diarrhoea	Lameness	Dyspnoea	Rectal swab	Serum	Tonsil	ADG (kg)	Mortality
DA 0.5%	0%^a^	0%^a^	0%^a^	7/30^a^	0/6^a^	0/30^a^	0.75^a^	0%
DA 0.15%	0%^a^	0%^a^	0%^a^	28/30^b^	0/6^a^	0/30^a^	0.64^b^	2%
(+) control	75%^b^	17%^a^	50%^b^	28/30^b^	2/6^b^	2/30^a^	0.65^b^	4%
*p*‐value	.03	.35	.03	<.0001	.11	.13	<.0001	NA

Note

Difference in superscripts (a/b) indicates a difference in significance of *p* < .05. Clinical scores are based on visual observations across the 6 pens in each room on days 6 and 15 post‐inoculation. Post‐mortem diagnostic data are based on results from 30 of 96 pigs from each room necropsied on day 15 post‐inoculation. Rectal swabs were tested for the presence of PEDV RNA, serum samples (pooled 5:1) were tested for the presence of PRRSV RNA, and tonsils were tested for the presence of SVA RNA. Performance data are summarized by room.

**Table 2b:** Summary of per cent positive pens and mean Ct values from feeder trough samples and oral fluid samples by virus type across treatment and control groups collected at day 6 and day 15 post‐inoculation (DPI) from Experiment 1

Treatment	Oral Fluid 6 DPI	Oral Fluid 15 DPI	Feed 6 DPI	Feed 15 DPI
PEDV				
DA 0.5%	0%^a^ (+)/Ct = 38.0^b^	17% (+)/Ct = 36.5	0% (+)/Ct = 38.0	100% (+)/Ct = 34.8
DA 0.15%	100% (+)/Ct s = 31.6	33% (+)/Ct = 34.9	83% (+)/Ct = 33.7	100% (+)/Ct = 32.2
(+) control	33% (+)/Ct = 32.8	50% (+)/Ct = 36.4	33% (+)/Ct = 33.6	100% (+)/Ct = 32.8
PRRSV				
DA 0.5%	0% (+)/Ct = 38.0	0% (+)/Ct = 38.0	0% (+)/Ct = 38.0	0% (+)/Ct = 38.0
DA 0.15%	0% (+)/Ct = 38.0	0% (+)/Ct = 38.0	0% (+)/Ct = 38.0	0% (+)/Ct = 38.0
(+) control	50% (+)/Ct = 28.0	50% (+)/Ct = 28.0	33% (+)/Ct = 26.5	0% (+)/Ct = 38.0
SVA				
DA 0.5%	33% (+)/Ct = 35.6	0% (+)/Ct = 38.0	33% (+)/Ct = 26.1	0% (+)/Ct = 38.0
DA 0.15%	50% (+)/Ct = 35.8	0% (+)/Ct = 38.0	17% (+)/Ct = 27.1	0% (+)/Ct = 38.0
(+) control	50% (+)/Ct = 31.4	0% (+)/Ct = 38.0	67% (+)/Ct = 33.8	0% (+)/Ct = 38.0

^a^ Percentage of positive pens per room based on 6 pens per room.

^b^ Mean Ct value across the number of pens containing positive samples.

### 3.3 Experiment 2 summary

Experiment 2 was conducted in January 2019. This experiment involved two products: (a) SalCURB^®^, a blend of aqueous formaldehyde and organic acids applied at a rate of 0.275% per ton of complete feed and (b) CaptiSURE™, a blend of medium‐chain fatty acids applied at a rate of 1.0% per ton of complete feed (Kemin Industries). Based on data summarized in Table 3a, PRRSV, PEDV and SVA infection occurred in the positive control group, along with clinical signs, poor performance (0.35 kg ADG) and elevated mortality (4%). In contrast, no evidence of viral RNA or clinical signs of disease were detected in the SalCURB^®^ or CaptiSURE™, groups. These groups also displayed improved health and performance with an increase in ADG of ≥120% as compared to controls (0.42 kg–0.43 kg versus. 0.35 kg, *p* < .0001) and numerically lower mortality (0%). No statistical differences were observed between the two products. Finally, PRRSV, PEDV and SVA RNA were detected in multiple feed trough samples and oral fluid samples from treatment and positive control groups throughout the study (Table 3b).

**Table 3a:** Summary of clinical scores, post‐mortem diagnostics and pig performance from Experiment 2

Treatment	Clinical scores			Post‐mortem diagnostics			Performance	
	Diarrhoea	Lameness	Dyspnoea	Rectal swab	Serum	Tonsil	ADG (kg)	Mortality
SalCURB^®^	0%^a^	0%^a^	0%^a^	0/30^a^	0/6^a^	0/30^a^	0.42^a^	0%
CaptiSURE™ 1.0%	0%^a^	0%^a^	0%^a^	0/30^a^	0/6^a^	0/30^a^	0.43^a^	0%
(+) control	100%^b^	17%^a^	100%^b^	5/30^b^	6/6^b^	13/30^b^	0.35^b^	4%
*p*‐value	.0001	.35	.0001	.005	.0001	<.0001	<.0001	NA

Note

Difference in superscripts (a/b) indicates a difference in significance of *p* < .05. Clinical scores are based on visual observations across the 6 pens in each room on days 6 and 15 post‐inoculation. Post‐mortem diagnostic data are based on results from 30 of 96 pigs from each room necropsied on day 15 post‐inoculation. Rectal swabs were tested for the presence of PEDV RNA, serum samples (pooled 5:1) were tested for the presence of PRRSV RNA, and tonsils were tested for the presence of SVA RNA. Performance data are summarized by room.

**Table 3b:** Summary of per cent positive pens and mean Ct values from feeder trough samples and oral fluid samples by virus type across treatment and control groups collected at day 6 and day 15 post‐inoculation (DPI) from Experiment 2

Treatment	Oral Fluid 6 DPI	Oral Fluid 15 DPI	Feed 6 DPI	Feed 15 DPI
PEDV				
SalCURB^®^	33% (+)^a^/Ct = 33.2^b^	0% (+)/Ct = 38.0	33% (+)/Ct = 32.5	0% (+)/Ct = 38.0
CaptiSURE™ (1.0%)	50% (+)/Ct = 34.9	0% (+)/Ct = 38.0	33% (+)/Ct = 33.7	0% (+)/Ct = 38.0
(+) control	33% (+)/Ct = 31.7	17% (+)/Ct = 36.1	17% (+)/Ct = 37.1	0% (+)/Ct = 38.0
PRRSV				
SalCURB^®^	67% (+)/Ct = 32.4	17% (+)/Ct = 36.9	33% (+)/Ct = 28.5	0% (+)/Ct = 38.0
CaptiSURE™ (1.0%)	100% (+)/Ct = 32.0	17% (+)/Ct = 36.1	33% (+)/Ct = 29.3	0% (+)/Ct = 38.0
(+) control	83% (+)/Ct = 32.8	100% (+)/Ct = 27.3	17% (+)/Ct = 34.7	100% (+)/Ct = 34.1
SVA				
SalCURB^®^	33% (+)/Ct = 26.2	0% (+)/Ct = 38.0	33% (+)/Ct = 26.1	0% (+)/Ct = 38.0
CaptiSURE™ (1.0%)	50% (+)/Ct = 27.5	0% (+)/Ct = 38.0	33% (+)/Ct = 27.1	0% (+)/Ct = 38.0
(+) control	33% (+)/Ct = 26.4	50% (+)/Ct = 30.7	67% (+)/Ct = 33.8	50% (+)/Ct = 35.0

^a^Percentage of positive pens per room based on 6 pens per room.

^b^Mean Ct value across the number of pens containing positive samples.

### 3.4 Experiment 3 summary

Experiment 3 was conducted in March 2019. This experiment involved four products and a positive control group. Products included (a) DaaFit^®^S (Archer Daniels Midland Company) a source of fatty acids, including lauric and myristic acids and glycerol monolaurate, applied at a 0.5% inclusion rate or a 0.3% inclusion rate per ton of complete feed, (b) Dominnate, a blend of three medium‐chain fatty acids applied at a 0.5% inclusion rate (Purina Animal Nutrition), (c) SalCURB^®^K2, (Kemin Industries), an organic acid blend, including formic acid, ammonium formate, propionic acid and lactic acid applied at a 0.275% inclusion rate, and (d) Finio^®^ (Anitox), a blend of propionic acid, trans‐2‐hexenal, (leaf aldehyde) and nonanoic acid (pelargonic acid) applied at a 0.2% inclusion rate per ton of complete feed. Based on data from Table 4a, RNA from all three viruses was detected in multiple tissue and environmental samples from the positive control group, along with evidence of clinical signs suggestive of all three diseases, poor performance (0.2 kg ADG) and elevated mortality (5%). In contrast, ADG improved by ≥160% as compared to controls (0.32 kg–0.35 kg versus 0.2 kg, *p* < .0001), with numerically lower mortality (0%) across all treatment groups. Detection of PEDV RNA in rectal swabs and SVA in tonsil samples, and all serum samples collected from the five treatment groups were PCR‐negative for PRRSV (Table 4b). Finally, PRRSV, PEDV and SVA RNA were detected in multiple feed trough samples and oral fluid samples from the treatment and positive control groups throughout the study.

**Table 4a:** Summary of clinical scores, post‐mortem diagnostics and pig performance from Experiment 3

	Clinical scores			Post‐mortem diagnostics Post‐mortem diagnostics			Performance	
	Diarrhoea	Lameness	Dyspnoea	Rectal swab	Serum	Tonsil	ADG (kg)	Mortality
Daafit^®^S 0.5%	17%^a^	0%^a^	0%^a^	8/30^ab^	0/6^a^	0/30^a^	0.35^a^	0%
Daafit^®^S 0.3%	17%^a^	17%^a^	0%^a^	12/30^b^	0/6^a^	10/30^b^	0.35^a^	0%
Dominnate	33%^a^	0%^a^	0%^a^	7/30^ab^	0/6^a^	0/30^a^	0.32^a^	0%
SalCURB^®^K2	17%^a^	17%^a^	0%^a^	8/30^ab^	0/6^a^	6/30^b^	0.35^a^	0%
Finio^®^	50%^ab^	17%^a^	0%^a^	3/30^a^	0/6^a^	9/30^b^	0.32^a^	0%
(+) control	100%^b^	100%^b^	100%^b^	23/30^c^	6/6^b^	6/30^b^	0.20^b^	5%
*p*‐value	.02	.004	<.0001	<.0001	<.0001	.0006	<.0001	NA

Note

Difference in superscripts (a/b) indicates a difference in significance of *p* < .05. Clinical scores are based on visual observations across the 6 pens in each room on days 6 and 15 post‐inoculation. Post‐mortem diagnostic data are based on results from 30 of 96 pigs from each room necropsied on day 15 post‐inoculation. Rectal swabs were tested for the presence of PEDV RNA, serum samples (pooled 5:1) were tested for the presence of PRRSV RNA, and tonsils were tested for the presence of SVA RNA. Performance data are summarized by room.

**Table 4b:** Summary of per cent positive pens and mean Ct values from feeder trough samples and oral fluid samples by virus type across treatment and control groups collected at day 6 and day 15 post‐inoculation (DPI) from Experiment 3

Treatment	Oral Fluid 6 DPI	Oral Fluid 15 DPI	Feed 6 DPI	Feed 15 DPI
PEDV				
DaaFit^®^S 0.5%	17% (+)^a^/Ct = 36.9^b^	17% (+)/Ct = 35.4	17% (+)/Ct = 35.6	0% (+)/Ct = 38.0
DaaFit^®^S 0.3%	17% (+)/Ct = 36.5	0% (+)/Ct = 38.0	17% (+)/Ct = 36.5	0% (+)/Ct = 38.0
Dominnate	17% (+)/Ct = 34.8	17% (+)/Ct = 36.3	0% (+)/Ct = 38.0	0% (+)/Ct = 38.0
SalCURB^®^K2	33% (+)/Ct = 35.1	0% (+)/Ct = 38.0	0% (+)/Ct = 38.0	0% (+)/Ct = 38.0
Finio^®^	33% (+)/Ct = 35.4	33% (+)/Ct = 37.5	33% (+)/Ct = 35.5	0% (+)/Ct = 38.0
(+) control	100% (+)/Ct = 32.1	100% (+)/Ct = 27.4	33% (+)/Ct = 36.5	17% (+)/Ct = 32.5
PRRSV				
DaaFit^®^S 0.5%	17% (+)/Ct = 32.5	0% (+)/Ct = 38.0	17% (+)/Ct = 30.8	0% (+)/Ct = 38.0
DaaFit^®^S 0.3%	17% (+)/Ct = 31.7	0% (+)/Ct = 38.0	17% (+)/Ct = 32.1	0% (+)/Ct = 38.0
Dominnate	17% (+)/Ct = 31.4	0% (+)/Ct = 38.0	0% (+)/Ct = 38.0	0% (+)/Ct = 38.0
SalCURB^®^K2	17% (+)/Ct = 32.1	0% (+)/Ct = 38.0	17% (+)/Ct = 32.1	0% (+)/Ct = 38.0
Finio^®^	33% (+)/Ct = 31.3	0% (+)/Ct = 38.0	33% (+)/Ct = 30.9	0% (+)/Ct = 38.0
(+) control	83% (+)/Ct = 31.5	100% (+)/Ct = 25.7	33% (+)/Ct = 29.2	67% (+)/Ct = 36.4
SVA				
DaaFit^®^S 0.5%	17% (+)/Ct = 27.2	0% (+)/Ct = 38.0	33% (+)/Ct = 32.1	0% (+)/Ct = 38.0
DaaFit^®^S 0.3%	33% (+)/Ct = 33.1	83% (+)/Ct = 31.6	83% (+)/Ct = 35.2	0% (+)/Ct = 38.0
Dominnate	17% (+)/Ct = 26.1	0% (+)/Ct = 38.0	67% (+)/Ct = 36.9	0% (+)/Ct = 38.0
SalCURB^®^K2	17% (+)/Ct = 26.5	100% (+)/Ct = 31.9	67% (+)/Ct = 34.3	17% (+)/Ct = 32.9
Finio^®^	33% (+)/Ct = 29.5	0% (+)/Ct = 38.0	50% (+)/Ct = 30.3	0% (+)/Ct = 38.0
(+) control	50% (+)/Ct = 33.5	17% (+)/Ct = 36.9	33% (+)/Ct = 34.2	0% (+)/Ct = 38.0

^a^ Percentage of positive pens per room based on 6 pens per room.

^b^ Mean Ct value across the number of pens containing positive samples.

### 3.5 Experiment 4 summary

Experiment 4 was conducted in November 2019. This experiment involved four products and a positive control group. Products included (a) VVC (DSM Nutritional Products, Heerlen, the Netherlands), a product comprised of pure benzoic acid and nature‐identical flavourings at either a 0.5% or 0.3% inclusion rate per ton of complete feed, (b) R2™ (Feed Energy, Pleasant Hill, IA), a combination of short‐, medium‐ and long‐chain fatty acids, applied at a 3.0% inclusion rate, (c) CaptiSURE™, a blend of medium‐chain fatty acids applied at a reduced rate of 0.5% per ton of complete feed, and (d) Guardian (Alltech), a blend of organic acids and essential oils at a 0.44% inclusion rate. Based on data from Table 5a, RNA from all three viruses was detected in samples from the positive control group, along with evidence of clinical signs suggestive of all three diseases, poor performance (0.14 kg ADG) and elevated mortality (10%). Average daily gain improved by > 357% across the treatment groups as compared to controls (0.50 kg–0.55 kg versus 0.14 kg, *p* < .0001), along with numerically lower mortality (≤1%) and reduced clinical signs. Despite the presence of viral RNA in post‐mortem samples from several treatments, all groups demonstrated improved health and performance as compared to controls. Finally, PRRSV, PEDV and SVA RNA were detected in multiple feed trough samples and oral fluid samples from the treatment and control groups throughout the study (Table 5b).

**Table 5a:** Summary of clinical scores, post‐mortem diagnostics and pig performance from Experiment 4

Treatment	Clinical scores			Post‐mortem diagnostics			Performance	
	Diarrhoea	Lameness	Dyspnoea	Rectal swab	Serum	Tonsil	ADG (kg)	Mortality
VVC 0.5%	0%^a^	0%^a^	0%^a^	0/30^a^	0/6^a^	0/30^a^	0.54^a^	1%
VVC 0.3%	0%^a^	0%^a^	0%^a^	0/30^a^	0/6^a^	0/30^a^	0.50^a^	1%
R2™	0%^a^	0%^a^	0%^a^	0/30^a^	0/6^a^	4/30^a^	0.53^a^	0%
CaptiSURE™0.5%	0%^a^	0%^a^	0%^a^	1/30^a^	0/6^a^	0/30^a^	0.52^a^	0%
Guardian	0%^a^	0%^a^	0%^a^	0/30^a^	4/6^b^	0/30^a^	0.55^a^	0%
(+) control	100%^b^	100%^b^	100%^b^	30/30^b^	6/6^b^	10/30^b^	0.14^b^	10%
*p*‐value	<.0001	<.0001	<.0001	<.0001	<.0001	<.0001	<.0001	NA

Note

Difference in superscripts (a/b) indicates a difference in significance of *p* < .05. Clinical scores are based on visual observations across the 6 pens in each room on days 6 and 15 post‐inoculation. Post‐mortem diagnostic data are based on results from 30 of 96 pigs from each room necropsied on day 15 post‐inoculation. Rectal swabs were tested for the presence of PEDV RNA, serum samples (pooled 5:1) were tested for the presence of PRRSV RNA, and tonsils were tested for the presence of SVA RNA. Performance data are summarized by room.

**Table 5b:** Summary of per cent positive pens and mean Ct values from feeder trough samples and oral fluid samples by virus type across treatment and control groups collected at day 6 and day 15 post‐inoculation (DPI) from Experiment 4

Treatment	Oral Fluid 6 DPI	Oral Fluid 15 DPI	Feed 6 DPI	Feed 15 DPI
PEDV				
VVC 0.5%	17% (+)^a^/Ct = 34.2^b^	50% (+)/Ct = 31.6	17% (+)/Ct = 26.9	17% (+)/Ct = 27.6
VVC 0.15%	17% (+)/Ct = 29.4	33% (+)/Ct = 32.9	17% (+)/Ct = 26.2	33% (+)/Ct = 30.1
R2™	50% (+)/Ct = 32.6	33% (+)/Ct = 30.3	17% (+)/Ct = 27.6	50% (+)/Ct = 32.8
CaptiSURE™ 0.5%	33% (+)/Ct = 30.2	33% (+)/Ct = 29.6	17% (+)/Ct = 27.4	33% (+)/Ct = 27.7
Guardian	50% (+)/Ct = 30.4	33% (+)/Ct = 30.6	50% (+)/Ct = 33.8	33% (+)/Ct = 31.9
(+) control	100% (+)/Ct = 27.5	100% (+)/Ct = 26.3	83% (+)/Ct = 32.1	100% (+)/Ct = 25.1
PRRSV				
VVC 0.5%	33% (+)/Ct = 27.5	50% (+)/Ct = 33.5	33% (+)/Ct = 27.5	17% (+)/Ct = 26.4
VVC 0.15%	33% (+)/Ct = 30.3	33% (+)/Ct = 33.1	33% (+)/Ct = 27.4	33% (+)/Ct = 30.6
R2™	33% (+)/Ct = 31.3	33% (+)/Ct = 32.1	33% (+)/Ct = 28.5	67% (+)/Ct = 32.8
CaptiSURE™ 0.5%	33% (+)/Ct = 30.9	33% (+)/Ct = 33.1	17% (+)/Ct = 25.1	33% (+)/Ct = 32.6
Guardian	50% (+)/Ct = 31.7	100% (+)/Ct = 30.9	33% (+)/Ct = 27.9	50% (+)/Ct = 33.0
(+) control	100% (+)/Ct = 27.3	100% (+)/Ct = 25.7	83% (+)/Ct = 32.5	100% (+)/Ct = 32.6
SVA				
VVC 0.5%	33% (+)/Ct = 34.2	17% (+)/Ct = 27.2	50% (+)/Ct = 32.9	17% (+)/Ct = 28.3
VVC 0.15%	33% (+)/Ct = 33.1	17% (+)/Ct = 28.9	50% (+)/Ct = 31.9	33% (+)/Ct = 31.0
R2™	50% (+)/Ct = 33.6	50% (+)/Ct = 29.9	33% (+)/Ct = 30.5	50% (+)/Ct = 32.0
CaptiSURE™ 0.5%	33% (+)/Ct = 32.9	17% (+)/Ct = 27.9	33% (+)/Ct = 30.9	50% (+)/Ct = 33.1
Guardian	17% (+)/Ct = 28.5	17% (+)/Ct = 30.1	50% (+)/Ct = 32.9	33% (+)/Ct = 31.4
(+) control	33% (+)/Ct = 31.0	50% (+)/Ct = 28.1	50% (+)/Ct = 30.1	67% (+)/Ct = 27.5

^a^ Percentage of positive pens per room based on 6 pens per room.

^b^ Mean Ct value across the number of pens containing positive samples.

### 3.6 Experiment 5 summary

Experiment 5 was conducted in February 2020. This experiment involved five products and a positive control group. Products included (a) pHorce (Anpario, Nottinghamshire, the UK) a liquid blend of formic acid and propionic acid on a mineral carrier at 0.3% inclusion rate per ton of complete feed, (b) DaaFit^®^PLUS, a blend of short‐chain fatty acids, including formic acid, propionic acid, acetic acid, sorbic acid and a blend of medium‐chain fatty acids including lauric acid, caprylic acid, and glycerol monolaurate at 0.5% inclusion (Archer Daniels Midland Company), (c) Dual Defender™, a blend of essential oils and prebiotic fibre applied at a 0.1% inclusion rate (Ralco Nutrition), (d) FURST PROTECT, a blend of emulsifying monoglycerides of medium‐chain fatty acids and essential oils and botanical extracts applied at a 0.4% inclusion rate (Furst McNess Company) and (e) Vigilex, a blend of oils, bacterial fermentation products, whey products, plant protein and natural flavourings applied at a 0.4% inclusion rate per ton of complete feed (Provimi North America). Based on data from Table 6a, RNA from all three viruses was detected in samples from the positive control group, along with evidence of clinical signs suggestive of all three diseases, poor performance (0.24 kg ADG) and elevated mortality (6%). Improved health and performance was seen in four of the five treatment groups (pHorce, DaaFit^®^PLUS, Dual Defender™ and FURST PROTECT). The ADG across all four groups increased by ≥216% as compared to controls (0.52 kg–0.59 kg versus 0.24 kg, *p* < .0001), along with <1% mortality and 0% incidence of clinical signs. All serum and tonsil samples from pigs consuming feed supplemented with pHorce, DaaFit^®^PLUS, Dual Defender™ and FURST PROTECT were PCR‐negative for PRRSV and SVA, respectively. PEDV RNA was detected in rectal swabs from four treatments but at significantly lower level than controls. In contrast, pigs consuming Vigilex‐treated feed had significantly lower ADG (0.28 kg) and elevated mortality (7%) compared to the other four treatments, along with a level of clinical signs and frequency of viral RNA detection in post‐mortem samples similar to the positive control group. Finally, PRRSV, PEDV and SVA RNA were detected in multiple feed trough samples and oral fluid samples from the treatment and positive control groups (Table 6b).

**Table 6a:** Summary of clinical scores, post‐mortem diagnostics and pig performance from Experiment 5

Treatment	Clinical scores			Post‐mortem diagnostics Post‐mortem diagnostics			Performance	
	Diarrhoea	Lameness	Dyspnoea	Rectal swab	Serum	Tonsil	ADG (kg)	Mortality
pHorce	0%^a^	0%^a^	0%^a^	9/30^a^	0/6^a^	0/30^a^	0.58^a^	1%
Daafit^®^PLUS	0%^a^	0%^a^	0%^a^	12/30^a^	0/6^a^	0/30^a^	0.52^a^	0%
Dual Defender™	0%^a^	0%^a^	0%^a^	12/30^a^	0/6^a^	0/30^a^	0.57^a^	0%
FURST PROTECT	0%^a^	0%^a^	0%^a^	14/30^a^	0/6^a^	0/30^a^	0.59^a^	0%
Vigilex	67%^b^	100%^b^	100%^b^	30/30^b^	4/6^a^	5/30^b^	0.28^a^	7%
(+) control	100%^b^	100%^b^	100%^b^	30/30^b^	6/6^b^	10/30^b^	0.24^b^	6%
*p*‐value	.0002	<.0001	<.0001	<.0001	<.0001	<.0001	<.0001	NA

Note

Difference in superscripts (a/b) indicates a difference in significance of *p* < .05. Clinical scores are based on visual observations across the 6 pens in each room on days 6 and 15 post‐inoculation. Post‐mortem diagnostic data are based on results from 30 of 96 pigs from each room necropsied on day 15 post‐inoculation. Rectal swabs were tested for the presence of PEDV RNA, serum samples (pooled 5:1) were tested for the presence of PRRSV RNA, and tonsils were tested for the presence of SVA RNA. Performance data are summarized by room.

**Table 6b:** Summary of per cent positive pens and mean Ct values from feeder trough samples and oral fluid samples by virus type across treatment and control groups collected at day 6 and day 15 post‐inoculation (DPI) from Experiment 5

Treatment	Oral Fluid 6 DPI	Oral Fluid 15 DPI	Feed 6 DPI	Feed 15 DPI
PEDV PEDV				
pHorce	0% (+)^a^/Ct = 38.0^b^	33% (+)/Ct = 30.6	50% (+)/Ct = 35.1	67% (+)/Ct = 30.8
Daafit^®^PLUS	0% (+)/Ct = 38.0	33% (+)/Ct = 30.0	33% (+)/Ct = 33.3	33% (+)/Ct = 32.7
Dual Defender™	0% (+)/Ct = 38.0	17% (+)/Ct = 31.5	33% (+)/Ct = 33.4	50% (+)/Ct = 30.6
FURST PROTECT	0% (+)/Ct = 38.0	33% (+)/Ct = 32.9	33% (+)/Ct = 33.9	50% (+)/Ct = 31.8
Vigilex	33% (+)/Ct = 33.8	100% (+)/Ct = 23.9	50% (+)/Ct = 34.2	100% (+)/Ct = 30.3
(+) control	100% (+)/Ct = 29.7	100% (+)/Ct = 22.8	67% (+)/Ct = 33.7	100% (+)/Ct = 29.4
PRRSV				
pHorce	0% (+)/Ct = 38.0	33% (+)/Ct = 26.4	17% (+)/Ct = 26.5	67% (+)/Ct = 27.4
Daafit^®^PLUS	0% (+)/Ct = 38.0	33% (+)/Ct = 28.2	17% (+)/Ct = 25.8	33% (+)/Ct = 24.9
Dual Defender™	0% (+)/Ct = 38.0	33% (+)/Ct = 32.3	0% (+)/Ct = 38.0	50% (+)/Ct = 26.1
FURST PROTECT	0% (+)/Ct = 38.0	33% (+)/Ct = 28.4	17% (+)/Ct = 25.4	33% (+)/Ct = 30.4
Vigilex	100% (+)/Ct = 30.2	100% (+)/Ct = 26.4	33% (+)/Ct = 30.4	100% (+)/Ct = 29.8
(+) control	100% (+)/Ct = 29.9	100% (+)/Ct = 25.9	33% (+)/Ct = 30.9	100% (+)/Ct = 24.6
SVA				
pHorce	0% (+)/Ct = 38.0	33% (+)/Ct = 31.9	17% (+)/Ct = 32.9	67% (+)/Ct = 30.7
Daafit^®^PLUS	0% (+)/Ct = 38.0	33% (+)/Ct = 34.5	17% (+)/Ct = 31.9	33% (+)/Ct = 31.8
Dual Defender™	0% (+)/Ct = 38.0	33% (+)/Ct = 32.9	33% (+)/Ct = 30.5	50% (+)/Ct = 32.0
FURST PROTECT	0% (+)/Ct = 38.0	17% (+)/Ct = 34.8	33% (+)/Ct = 30.9	50% (+)/Ct = 30.4
Vigilex	17% (+)/Ct = 33.9	33% (+)/Ct = 33.9	50% (+)/Ct = 32.9	100% (+)/Ct = 32.4
(+) control	50% (+)/Ct = 32.8	50% (+)/Ct = 29.1	50% (+)/Ct = 30.1	67% (+)/Ct = 30.7

^a^ Percentage of positive pens per room based on 6 pens per room.

^b^ Mean Ct value across the number of pens containing positive samples.

### 3.7 Meta‐analysis

**Figure 1:**
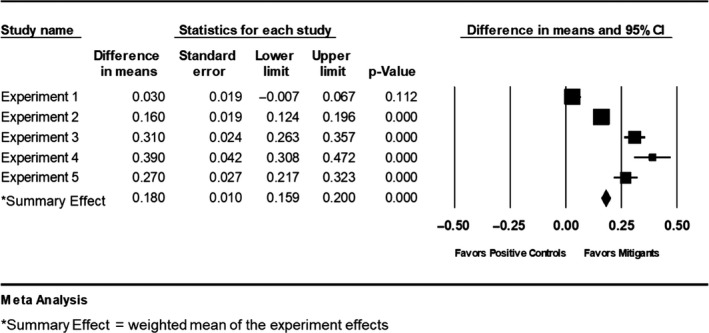
legend: Forest plot outlining the difference in the means, standard errors, 95% confidence intervals and *p*‐values from the five experiments. The summary effect reports the weighted average difference, standard error, and 95% confidence interval, and *p*‐value across all five experiments. Results indicated that the summary effect was 0.18 kg/day greater average daily gain, significantly favouring mitigated treatments (95% CI 0.16–0.20, *p* < .0001)

The results of the meta‐analysis (Figure 1) indicated that the summary effect was 0.18 kg/day greater average daily gain, significantly favouring mitigated treatments (95% CI 0.16–0.20, *p* < .0001), as compared to non‐mitigated controls.

## 4 DISCUSSION

The purpose of this study was to design a model simulating large‐scale commercial swine production to evaluate whether multiple chemically diverse feed additives could reduce the risk of viral‐contaminated feed. The study was based on the hypothesis that the use of an additive would improve pig health and performance, as compared to animals on non‐supplemented diets. To address this hypothesis, we employed an ‘ice‐block’ challenge involving three significant viruses of pigs, multiple metrics to measure additive efficacy, and large populations of animals housed under conditions representative of commercial production. Under the conditions of this study, it appeared that the majority (14 of 15) of products significantly improved pig health and performance as compared to pigs raised on non‐mitigated diets. For example, treatment of contaminated feed with 10 out of 15 products (Activate DA at 0.5%, SalCURB^®^, CaptiSURE™ at 1.0% and 0.5%, R2™, Guardian, DaaFit^®^PLUS, pHorce, VVC at 0.5% and 0.3%, Dual Defender™ and FURST PROTECT) led to no signs of clinical disease and a mortality level of ≤1%. This observation was further strengthened by the meta‐analysis which indicated that ADG across the five treatment groups was significantly greater (*p* = .000) as compared to control groups (Figure 1). It is unknown whether this would have changed had the study been conducted for a longer period; however, as we look at the data across treatment groups and controls during the five 15‐day study period, the differences are striking.

These results raise questions regarding the mechanism of action. It is possible that the products are ameliorating the disease at the level of the virus through a reduction in viral load and/or viability, or at the level of the pig through enhancement of the immune system, adjustment to the gut environment, manipulation of the microbiome or by some other mode. As this observation was consistent across a diverse portfolio of products, that is monovalent and multivalent organic acid products; short‐, medium‐ and long‐chain fatty acid blends; monoglycerides of fatty acids; formaldehyde‐based products; and essential oils (Table 1), there appears to be great opportunity for future research in this area. Furthermore, several of the products tested such as CaptiSURE™ (1.0% and 0.5%), DaaFit^®^S (0.5% and 0.3%) and VVC (0.5% and 0.3%) seemed to perform well at different inclusion rates, along with Dual Defender™ which performed well at an inclusion rate of 0.1%, is promising as it pertains to the economics of using additives in this manner.

An important component of the study is the viral challenge model. We used an ice block, containing equivalent concentrations of significant pathogens to simulate a point of contamination, that is a ‘hot spot’, at the level of the feed bin following delivery of feed to the farm, as opposed to the widespread dispersion of a pathogen that could occur following the mixing of a contaminated ingredient at the mill. Based on the metrics used in the study, this approach consistently delivered all three viruses to the treatment and control groups, including the first evidence of the transmission of PRRSV 174 following consumption of viral‐contaminated feed via natural feeding behaviour. The success of the ice‐block approach may have been enhanced by the time of year, as these experiments were conducted during cold weather which may have enhanced viral viability in the feed. This is supported by the fact that an experiment conducted during warmer weather (early October 2019), was not successful, as positive control pigs did not become infected to PEDV or SVA (Dee, unpublished data). One criticism of this approach could be that the levels of virus in the ice block were excessive and therefore not representative of the actual viral load in feed under commercial conditions. While this is a valid point, information on the actual level of virus contamination in commercial feed is not currently available; therefore, we based the concentration used on data from field samples of feed naturally contaminated with PEDV, along with multiple publications involving experimental inoculation of feed ingredients using this same amount (Dee et al., 2018; Dee, Clement, et al., 2014; Stoian et al., 2019, 2020). Surprisingly, when the viral load used in this study was calculated per gram of feed, the quantity appeared to be relatively small (7.69 v 10° 50% tissue culture infectious doses per gram of feed).

Another important aspect of the study design was the collection of multiple samples post‐challenge to document viral entry to the animal rooms via sampling the feed trough, followed by exposure of the animals via sampling oral fluids. Based on the data from Tables 2b through 6b, all three viruses consistently entered the treatment and control rooms via the feed and that viral RNA was subsequently detected in the oral cavities of pigs. It was interesting that the time between challenge and detection seemed to vary across the five experiments. For example, samples were PCR‐positive at day 6 post‐inoculation in experiments one through three, suggesting early exposure post‐challenge, in contrast to detection at both day 6 and day 15 post‐challenge (experiment four), or detection late in the study period (day 15, experiment five). However, due to the limited number of sampling days in each experiment, viral exposure could have occurred at other times and gone undetected.

As with all studies, this one had numerous acknowledged strengths and limitations. A major strength was the challenge model. This was a new approach, which not only provided a consistent means to deliver viral challenge to pigs via feed, but was also sensitive enough to detect an ineffective feed additive, that is Vigilex used in experiment 5. Other strengths include varying inclusion rates, different forms of additives (liquid or dry), use of multiple metrics to measure exposure and infection, and the testing of a diverse portfolio of product chemistries. Limitations include a design which only allowed for a single replicate per treatment, a limited number of sampling days per experiment, the inability to standardize pig age and weight on arrival across each experiment, the inherently variable delivery of virus due to feed bin flow, mixing of feed, natural feeding behaviour, and the decision to necropsy only 30 animals at the end of each experiment. This latter point is particularly important, since by only sampling a subset of animals in each treatment, one cannot confirm whether the entire population was truly negative or whether the sample size was incapable of detecting low levels of infection. Finally, the detection of viral RNA in feed samples and in some cases, detection of viral infection of pigs post‐consumption, indicates that while they seem to improve performance, additives do not appear to ‘sterilize’ the feed, which should be clearly communicated to manage expectations.

In closing, under the conditions of this study, the use of multiple feed additives significantly improved the health and performance of pigs as compared to animals on non‐supplemented diets. Based on the information from this project, the swine industry now has access to a list of validated products for use in a feed biosecurity programme. However, while these data are promising, there is still more work to be done. First of all, it is important to note that the products tested in this study do not have label approval claiming efficacy against viruses; however, several companies are working with the US Food and Drug Administration to rectify this situation. Secondly, while this study did not address foreign animal diseases, this work has stimulated interest in the ability of feed additives to mitigate the risk of ASFV in feed and studies are underway in certified facilities. Finally, it is hoped that the information from this project will motivate the swine industry, the veterinary profession and federal agencies to work together to develop a national programme of feed biosecurity involving the use of validated feed additives. Based on the growing body of scientific evidence in support of the risk of feed ingredients for the global spread of viral diseases, time is of the essence.

## CONFLICT OF INTEREST

Dr. Dee declares a conflict of interest as he played a role in development of the Guardian product and Pipestone has an ongoing financial interest in the sale of the product. Dr. Cochrane is a patent‐holder for a medium‐chain fatty acid product that is being licensed by a commercial feed additive company.

## ETHICAL APPROVAL

All animals involved in the study were managed in accordance with the institutional animal care and use guidelines observed by the investigators ethical review board (Pipestone Applied Research IACUC trial numbers 2018‐5, 2019‐10 and 2020‐02).

## Data Availability

All data from this study were made available in the paper.
